# Development of Bacterial Cellulose Biocomposites Combined with Starch and Collagen and Evaluation of Their Properties

**DOI:** 10.3390/ma14020458

**Published:** 2021-01-19

**Authors:** Silmar Baptista Nunes, Katharine Valéria Saraiva Hodel, Giulia da Costa Sacramento, Pollyana da Silva Melo, Fernando Luiz Pellegrini Pessoa, Josiane Dantas Viana Barbosa, Roberto Badaró, Bruna Aparecida Souza Machado

**Affiliations:** 1 PPG GETEC, University Center SENAI CIMATEC, National Service of Industrial Learning, SENAI CIMATEC, Salvador 41650-010, Brazil; silmar@fieb.org.br (S.B.N.); fernando.pessoa@fieb.org.br (F.L.P.P.); josianedantas@fieb.org.br (J.D.V.B.); badaro@fieb.org.br (R.B.); 2 SENAI Institute of Innovation (ISI) in Health Advanced Systems (CIMATEC ISI SAS), University Center SENAI/CIMATEC, SENAI CIMATEC, Salvador 41650-010, Brazil; katharine.hodel@fbter.org.br (K.V.S.H.); giuliacs@hotmail.com (G.d.C.S.); 3 Department of Materials, University Center SENAI CIMATEC, National Service of Industrial Learning, Salvador 41650-010, Brazil; pollyanam@fieb.org.br

**Keywords:** bacterial cellulose, starch, collagen, biopolymers, wound dressing

## Abstract

One of the major benefits of biomedicine is the use of biocomposites as wound dressings to help improve the treatment of injuries. Therefore, the main objective of this study was to develop and characterize biocomposites based on bacterial cellulose (BC) with different concentrations of collagen and starch and characterize their thermal, morphological, mechanical, physical, and barrier properties. In total, nine samples were produced with fixed amounts of glycerol and BC and variations in the amount of collagen and starch. The water activity (0.400–0.480), water solubility (12.94–69.7%), moisture (10.75–20.60%), thickness (0.04–0.11 mm), water vapor permeability (5.59–14.06 × 10^−8^ g·mm/m^2^·h·Pa), grammage (8.91–39.58 g·cm^−2^), opacity (8.37–36.67 Abs 600 nm·mm^−1^), elongation (4.81–169.54%), and tensile strength (0.99–16.32 MPa) were evaluated and defined. In addition, scanning electron microscopy showed that adding biopolymers in the cellulose matrix made the surface compact, which also influenced the visual appearance. Thus, the performance of the biocomposites was directly influenced by their composition. The performance of the different samples obtained resulted in them having different potentials for application considering the injury type. This provides a solution for the ineffectiveness of traditional dressings, which is one of the great problems of the biomedical sector.

## 1. Introduction

The last five decades have witnessed tremendous growth in the field of biomaterial science and engineering because of vast investments in the development of new products, including wound dressings [1]. Different materials have been analyzed to replace the traditional dressings for an effective treatment since most of the available dressings are used inappropriately (without considering the needs of each type of lesion), which can impair the wound healing process [2,3], especially in cases where the adhesive dressings damage the skin when removed, generating focal points of contamination [4]. In addition, the dressings directed to the treatment of specific lesions such as bedsores and burns have a high commercial cost [5,6]. Wound healing refers to the intrinsic and complex process because it involves cellular and biochemical phenomena, which is initiated from the rupture of the anatomical structure of the skin as well as the loss of its function, and aims to restore the integrity of skin tissue [2,7]. To recover its integrity, it is important that the place where the injury occurred be covered by a wound dressing to minimize the loss of its functions and assisting the process of tissue regeneration [8,9]. It is also known that a satisfactory material that covers the wound to prevent microbiological contamination and promotes a suitable environment for tissue regeneration is required for effective wound healing [10]. Hence, biomaterials such as polysaccharides (glycans) [11,12,13,14] and proteins [15,16] present an interesting alternative for this application due to their intrinsic properties that are considered essential for a dressing such as biocompatibility, non-toxicity, ability to adsorb bioactive molecules, and biodegradability [17].

As a result of this strong research, several materials have been suggested as potential candidates for biomedical application. Among the materials reported so far, bacterial cellulose (BC) has the possibility of use in different sectors of industry and has been widely studied in the health area because of its biocompatibility [18]. The obtaining of the BC is accomplished through the cultivation of different Gram-negative bacteria such as *Gluconacetobacter*, the most efficient BC producer [19,20,21], *Agrobacterium*, *Aerobacter*, *Azobacter*, besides other genera less used for this purpose such as *Rhizobium*, *Pseudomonas*, *Alcaligenes*, and *Enterobacter* [22,23]. These biopolymers consist of linear chains of covalent bonding chains β(1,4) between D-glucose subunits (β-1,4-glucan chains), forming bundles in the form of ribbons (microfibrils), which have various sizes and are arranged randomly, creating a porous structure [21,24]. The absence of lignin and hemicellulose (high purity) and organized physical structure provides BC with unique properties such as high crystallinity, thermal stability, and mechanical strength, which results in a performance superior to cellulose of plant origin [25]. In addition, other important properties of the BC are its high water-absorbing capabilities, being able to form hydrogels, biocompatibility (non-genotoxic and non-cytotoxic), besides having a slow degradation [21,26]. Several studies have proposed the use of BC with other polymers or molecules, resulting in the development of a new material with optimized properties aimed at its application as a wound dressing, through the addition of silver nanoparticles [27], chitosan [28], zinc oxide [29], titanium dioxide [30], collagen [31], and starch [32]. Recent studies have shown that the changes in the physical structure of BC using chemical modifiers improve the biological properties of the biomaterial, confirming its potential as an alternative material to develop an environmentally-friendly and biocompatible wound dressing, which promotes tissue regeneration [33,34].

Collagen is the major protein present in the extracellular matrix (ECM) and, as one of its main functions, acts as a support for connective tissues, being then responsible for maintaining the structure of the skin, blood vessels, bones, cartilage, tendons, and ligaments [35,36]. Collagen performs its functions through its interaction with the cells of the connective tissue and, from this interaction, acts to regulate different cellular events such as migration, anchoring, differentiation, proliferation, and survival [36]. Thus, collagen is an ECM component that can promote wound healing by stimulating myofibroblastic differentiation (cells capable of promoting and maintaining the inflammatory response to injury) and fibroblastic proliferation [8]. Collagen-based wound dressings have advantages when compared to other products because they are practical, since their physical structure is simple, homogeneous, and of abundant availability [36]. Considering the area of tissue engineering, BC-collagen composites have been synthesized mainly through in situ production strategies [37]. These composites exhibit better properties than pure BC such as improved mechanical properties and biocompatibility [38,39]. In addition to collagen, adding starch to the cellulose matrix also improves the mechanical properties and biocompatibility of the composite [32]. Starch is the second largest source of biomass worldwide, staying behind only cellulose (vegetal and bacterial), and a very important renewable resource in sustainable societies [40]. The pharmaceutical and biomedical sector has increased the use of starch in the last few decades due to different advantages including its natural and renewable source of obtaining and easy access due to the high abundance of raw material, which impacts the reduction of obtaining costs, besides its biodegradable and biocompatible nature [41].

Wound dressings are an important segment of the wound management market [42]. The global market for wound dressing is estimated to exceed $11 billion by 2025 from $7.0 billion in 2020, particularly due to the rising aging population and, consequently, the increased probability of chronic and surgical wound treatment as well as injuries of sudden onset. [43]. Thus, the range of available wound dressings based on biomaterials is expanding rapidly [44]. Different companies around the world produce biopolymer-based dressings in the form of hydrogels, hydrocolloids, alginates, foams, and films [45,46]. For example, a Brazilian company, BioFill Produtos Bioetecnologicos (Curitiba, Brazil) has developed Biofill, a BC-based wound dressing to be used for treating burns and ulcers as temporary artificial skin [47]. Another Brazilian company, Bionext, produces a BC product that regulates water moisture, promotes cell metabolism, and protects wounds from external microorganisms on a large scale. The European companies Coloplast (Humlebaek, Denmark) and Les Laboratoires Brothier (Nanterre, France) have developed alginate-based dressings Biatain and Algosteril, respectively, for the treatment of diabetic foot ulcers [46]. It is important to highlight that one of the great challenges associated with the development of modern wound dressings is the determination of an economic and scalable productive cycle, since the traditional dressings available in the market are inexpensive [48]. Therefore, it is important that inexpensive new products are developed using easily available biopolymers with optimized properties.

Based on the intrinsic properties of BC, collagen, and starch as well as the potential for application of biocomposites resulting from the combination of these biomaterials, this study aimed to develop and characterize biocomposites based on BC with different concentrations of collagen and starch and characterize them with respect to their thermal, mechanical, morphological, and physical and barrier properties, with the aim of their potential application as wound dressings.

## 2. Materials and Methods

**Figure 1:**
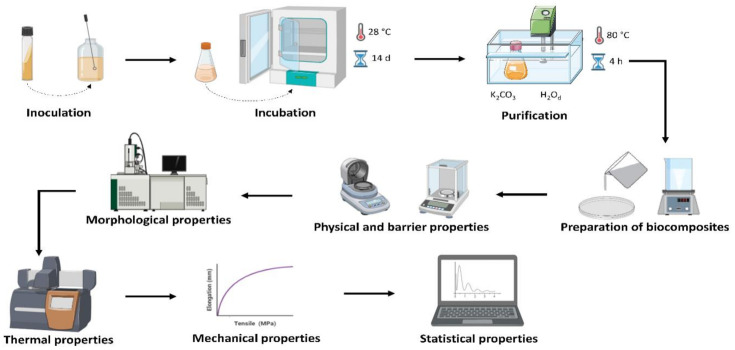
The main steps of the methodology used for bacterial cellulose (BC) and BC–collagen–starch biocomposite production and characterization. Created via BioRender.com.

Figure 1 illustrates the main steps of the methodology used to produce and obtain the BC membranes and BC–collagen–starch biocomposites as well as the characterization tests that have been applied.

### 2.1. Bacterial Cellulose (BC) Production and Purification

The *Glucanoacetobacter hansenii* ATCC23769 strain, obtained from the Tropical Cultures Collection (CCT)-André Tosello Foundation (São Paulo, SP, Brazil), was used to obtain BC by static fermentation. The culture medium for inoculum preparation and BC membrane formation had the following composition: 50 g·L^−1^ glucose, 5 g·L^−1^ yeast extract, 3 g·L^−1^ peptone, and 2 g·L^−1^ potassium phosphate (KH_2_PO_4_) [49]. The culture media were sterilized (121 °C, 15 min) by steam sterilization and incubated at 30 ± 2 °C. Fourteen days after strain inoculation, the BC membrane produced at the air–medium interface was obtained. Then, the BC membranes underwent the purification process through alkaline treatment with potassium carbonate (K_2_CO_3_). For this purpose, the membranes were washed twice with distilled water at 80 °C for 1 h to remove impurities from the culture medium and then were treated with 0.3 mol·L^−1^ K_2_CO_3_ aqueous solution at 80 °C for 1 h. After alkaline treatment, the BC membranes were washed with distilled water until a neutral pH (between 6.8 and 7) was obtained [49]. The purified membranes were stored at 4 °C in deionized water until further use.

### 2.2. Preparation of the Biocomposites

The purified BC was crushed in a multiprocessor and homogenized until a gel was obtained, and later used in the production of the biocomposites. Nine formulations (Table 1) were prepared using different cassava starch (16.40% amylose and 83.60% amylopectin; Amafil; Paraná, Brazil) and hydrolyzed collagen (Flora 7 Ervas; São Paulo, Brazil) contents, with a fixed value of BC (50% m·v^−1^) and glycerol (Synth; São Paulo, Brazil; 0.6% m·v^−1^). The biocomposites were produced by the casting technique [50] with gradual heating of the formulations up to 70 °C/60 rpm (C-MAG HS7; IKA; Staufen, Germany) for 20 min. Then, 45 g of each mixture was weighed in polystyrene Petri dishes and dehydrated in a drying oven at 40 ± 2 °C under airflow (Q314M222; Quimis; Diadema, Brazil) for 20–24 h. Figure S1 shows the steps involved in obtaining F1–F9 samples with real images. Before their characterization, pure BC and BC–collagen–starch biocomposites were stored in a desiccator containing a saturated solution of sodium chloride under ambient conditions of 23 ± 2 °C and relative humidity of 60% for 10 days.

### 2.3. Biocomposite Characterization

#### 2.3.1. Moisture, Total Solids (TS), and Water Activity (a_w_)

The total moisture and solid content of the pure BC and BC–collagen–starch biocomposites were determined using an infrared scale (Shimadzu, MOC-120H, Kyoto, Japan), which allowed the sample temperature to reach 105 °C through the emission of radiation. The weight loss (%) was evaluated as a function of the initial weight of the sample using the equipment software equation, determining the moisture and TS contents [51]. The a_w_ of the pure BC and biocomposites was analyzed in Decagon (Lab Master aw; Novasina; Lachen, Switzerland) at 25 °C using electrolytic cell CM-2. The “actual balance” [a_w_ = moisture in the balance sheet = actual balance (%)/100] was evaluated using the equipment software equation [52]. All analyses were performed in triplicate.

#### 2.3.2. Water Solubility (WS) and Water Vapor Permeability (WVP)

The WS of pure BC and BC–collagen–starch biocomposites was determined according to the assay presented by Moura et al. [53], where circular biocomposite specimens (2-cm in diameter) were weighed and then placed in a flask with 50 mL distilled water. The flask with the specimens were shaken for 24 h in an incubator with orbital shaker (MA420; Marconi; Piracicaba, Brazil) at room temperature (25 °C) and under agitation of 130 rpm. After this period, the specimens were dried at 105 °C for 24 h in a drying oven with forced air circulation (Q314M222; Quimis; Diadema, Brazil) to determine their final mass. The WS was determined in percentage according to Equation (1).

WS = ((m_0_ − m_1_))/m_1_ × 100 (1)

where WS is the solubility in water (%); m_0_ is the initial mass (g) of the specimens; and m_1_ is the dry mass (g) of the specimens after of contact with distilled water (solubilization).

The WVP was determined by the gravimetric method proposed by ASTM 96-00x with some modifications [54]. Circular specimens of pure BC and biocomposites (5 cm) were placed in permeation capsules containing silica gel (approximately 15 g, 0% relative humidity) and maintained in desiccators containing a saturated sodium chloride solution (75% relative humidity, 25 °C) for seven days. Every 24 h, the capsules containing the specimens were weighed to monitor the weight variation and the values obtained were plotted as a function of time. Thus, WVP was calculated by means of linear regression between the points of mass loss, according to Equation (2). All analyses were performed in triplicate.

WVP = (g × x)/(t × A × ΔP) (2)

where WVP is the water vapor permeability (g·mm^−1^·m^−2^·d^−1^·kPa^−1^); g is the pure BC or BC–collagen–starch biocomposite mass gain; x is the mean pure BC or BC–collagen–starch biocomposite thickness (mm); t is the total time (h); ΔP is the vapor pressure difference of the environment containing the silica gel (kPa at 25 °C) and pure water (3167 kPa at 25 °C) (g·t^−1^); and A is the permeation area (m^2^).

#### 2.3.3. Opacity and Grammage

The apparent opacity of the pure BC and BC–collagen–starch biocomposites was determined using a UV–Vis spectrophotometer (700 PLUS; FEMTO; São Paulo, Brazil), where the specimens were cut at rectangles and adhered to the internal wall of the quartz bucket, avoiding the trapping of air bubbles. Under these conditions, the opacity was measured at 500 nm [55]. The study by Almeida et al. was also used for grammage determination [56], where the grammage was calculated by the ratio between the mass of pure BC and biocomposites, determined from analytical balance weighing, and the specimen area (2 cm^2^). The opacity and grammage analyses were performed in triplicate.

#### 2.3.4. Thickness and Mechanical Properties

A flat-tip digital micrometer (Ip40; Digimess; São Paulo, Brazil) at a resolution of 0.001 mm was used to determine the thickness of the pure BC and BC–collagen–starch biocomposites. Thus, the thickness was evaluated by determining the average thickness of 10 quantifications in random positions of each respective specimen. The mechanical properties were analyzed using a texture analyzer (CT310k; Brookfield; Phoenix, AZ, USA) according to ASTM D-882, with adaptations [57]. Seven specimens (100 mm × 25 mm) were analyzed and conditioned under 58% relative humidity (RH) for 48 h at 25 °C. The samples were adjusted to the test points of the equipment (TA3/100 and TA/TPB) at an initial distance of 50 mm and pulled at a speed of 0.8 mm∙s^−1^. The properties determined were maximum tensile strength at break (MPa) and elongation at break (%).

#### 2.3.5. Swelling Rate (SR) and Water Release Rate (WRR)

For determining the swelling rate, three specimens (triplicate) of each biocomposite were made in the form of square membranes (15 mm side), weighed, and immersed separately in deionized water at room temperature (25 °C) for 6 h. Then, the samples were weighed after gently cleaning the surface using paper towels at certain intervals (1, 5, 10, 30, 60, 90, 120, 150, and 180 min) until constant weight was achieved. The degree of swelling was measured as the percentage of the initial increase in pure BC and BC–collagen–starch composite weight that occurred after swelling in water [58].

The capacity to release water from pure BC and BC–collagen–starch biocomposites was evaluated according to Ul-Islam et al. [59], with adaptations. The samples F1–F9 were cut into circular specimens (2 cm in diameter) to determine their initial dry weight (W_o_). The specimens were then placed in flasks containing deionized water and were maintained for 48 h. After this period, the swollen specimens were then rapidly dried with cellulose filter paper to remove excess water from the surface and placed on open petri dishes at 25 °C. The specimens were removed from the plates every 24 h to be weighed (W_w_), totaling four points of analysis or 96 h. The WRR was calculated by Equation (3). The analyses were performed in triplicate.

WRR (%) = (W_o_ − W_w_)/(W_o_) × 100 (3)

where WRR is the water retention rate in percent; W_o_ is the weight after immersion in deionized water and during the drying period at room temperature; and W_w_ is the initial weight of the dry membrane.

#### 2.3.6. Scanning Electron Microscopy (SEM)

Scanning electron microscopy (SEM, BX-51; Olympus; Tokyo, Japan) was used to evaluate the surface morphology and elements of the pure BC and BC–collagen–starch biocomposites. The preparation of the samples for this morphological analysis was performed as proposed in the study of Machado et al. [60], where the samples were manually fixed with tweezers (PELCO1 Tweezers) on an aluminum metallic structure using a carbon double-sided tape, known as stubs. Then, the metallization stage of the sample with gold was performed in a Balzers Sputter coater (SCD 50; BAL-TEC; Grand Island, NE, USA). The stubs containing the metallic samples were stored in storage boxes and double sealed with Parafilm (PARAFILM1 M) for moisture control. The samples were analyzed at 250 magnification (voltage, 15 kV; working distance, 30 mm; point size 50; HV vacuum mode).

#### 2.3.7. Thermogravimetric Analysis (TGA)

Thermogravimetric analysis (TGA) of pure BC and BC–collagen–starch biocomposites was performed on a Q50 thermogravimetric analyzer (TA Instruments; New Castle, DE, USA). TGA analysis was performed using about 6 mg of each sample that was subsequently placed in a platinum crucible. The sample recipient was placed in the thermogravimetric analyzer and subjected at a heating rate of 10 °C·min^−1^ from 25 to 900 °C under a nitrogen flow (30 mL·min^−1^). [61]. The results of the TGA were expressed as a percentage of mass loss (%)/temperature (°C) and differential thermogravimetry (DTG) curves were prepared from the TGA data.

### 2.4. Statistical Analysis

The results obtained related to the characterization of biocuratives were analyzed for variance (ANOVA) at 95% significance, and the results that present significant differences between treatments were differentiated by Tukey’s test. Assistat software (Version 7.7 beta) was used to analyze the results [62]. Principal component analysis (PCA) was performed using PAST (Paleontological Statistics; Oslo, Norway) version 3.26, developed by Øyvind Hammer, with the means of the characterization analyses [moisture (M), TS, a_w_, water solubility (WS), WVP, opacity (O), grammage (G), thickness (T), elongation at break (E), and tensile strength (MT)] to obtain the correlation between the produced samples and their properties. As they presented different units of measurement, the data concerning the characterization tests cited were normalized in the range of 0 to 1, and after standardization, the PCA was carried out.

## 3. Results

### 3.1. Visual Appearance of Pure BC and BC–Collagen–Starch Biocomposites

**Figure 2:**
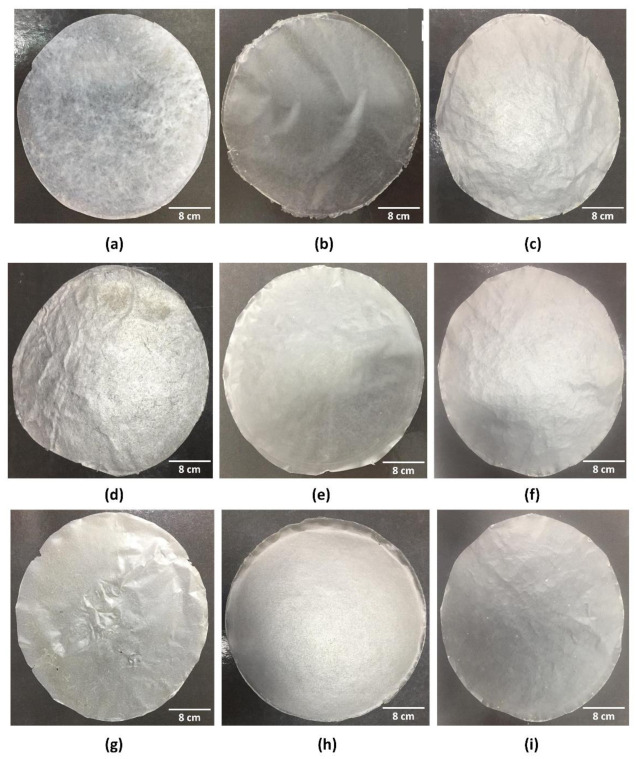
Visual appearance of the pure bacterial cellulose (BC; control) and BC–collagen–starch biocomposites. (**a**) F1, (**b**) F2, (**c**) F3, (**d**) F4, (**e**) F5, (**f**) F6, (**g**) F7, (**h**) F8, and (**i**) F9.

In total, eight different biocomposites based on BC, collagen, and starch and the control (pure BC–F1 sample) were produced according to Table 1. Figure 2 shows the physical appearance of the control (F1) and biocomposites (F2–F9) after complete evaporation of the solvent (water in this case). The formed biomaterials (pure BC and BC–collagen–starch biocomposites) were easily removed from the surface of the Petri dish, without rupturing its structure. Thus, all nine formulations were easy to handle and removal from the Petri dish did not compromise the specimens for the characterization analyses. The addition of other polymers in the cellulose matrix altered the physical appearance, since F1, which only had cellulose in its composition, was opaque with prominent cellulose fibers (Figure 1a). After adding collagen and starch, regardless of the biocomposite composition, the samples were transparent in appearance (Figure 1b–h).

### 3.2. Physical and Barrier Properties of Pure BC and BC–Collagen–Starch Biocomposites

**Figure 3:**
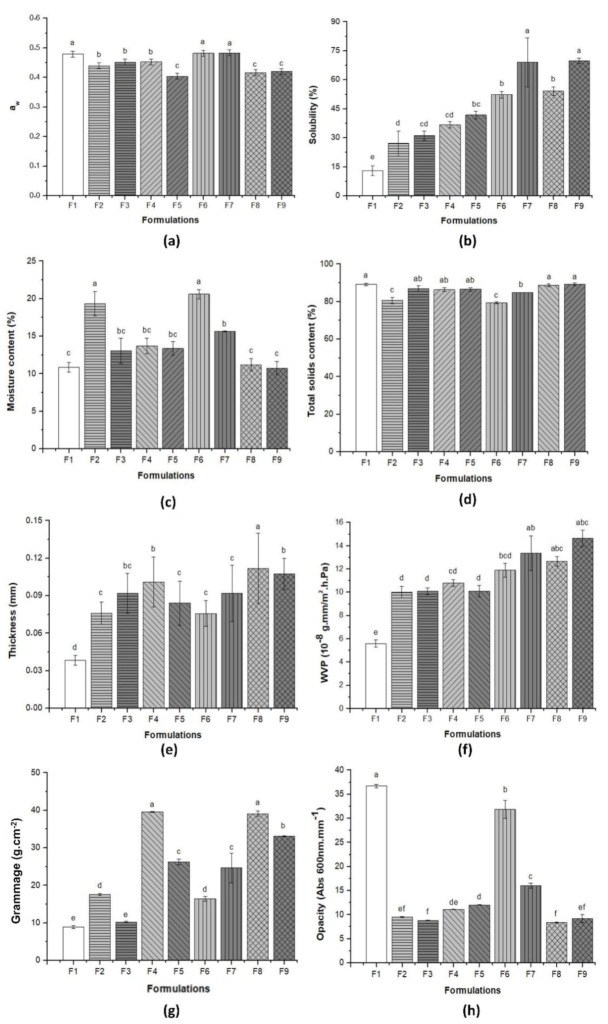
Physical and barrier properties of pure bacterial cellulose (BC, F1) and BC–collagen–starch biocomposites (F2–F9). (**a**) Water activity (a_w_) content; (**b**) Water solubility; (**c**) Moisture content; (**d**) Total solids content; (**e**) Thickness; (**f**) Water vapor permeability; (**g**) Grammage and (**h**) Opacity. Bars followed by the same letters were not significantly different at *p* < 0.05 according to Tukey’s test with 95% confidence.

Figure 3 and Table 2 show the results related to the physical and barrier properties of pure BC and the BC–collagen–starch biocomposites. The a_w_ of the nine samples varied between 0.400 ± 0.01 (F5) and 0.480 ± 0.01 (F1, F6, and F7), with significant differences between them (*p* > 0.05) (Figure 3a and Table 2). It is important to note that F1, F5, and F6, which had the highest a_w_, did not have starch, indicating that starch can influence a_w_. After adding collagen and/or starch in the cellulose matrix, the WS increased with significant difference between the samples, with the lowest in F1 (12.94 ± 2.58%) and the highest in F9 (69.76 ± 1.43%), which had collagen and starch besides BC (Figure 3b and Table 2). F7, F8, and F9, with the highest collagen content (2.00 g), had the highest WS (69.67 ± 12.7%, 54.12 ± 2.21%, and 69.76 ± 1.43%, respectively). However, F8, F9, and F1 had the lowest moisture (11.19 ± 0.81%, 10.75 ± 0.89%, and 10.88 ± 0.64%, respectively), while F6 had the highest moisture (20.60 ± 0.69%) (Figure 3c and Table 2). The TS content varied between 79.4 ± 0.69% (F6) and 89.25 ± 0.89% (F9), with a significant difference (*p* > 0.05) (Figure 3d and Table 2). The TS content was inversely proportional to the moisture content.

The thickness of the pure BC and biocomposites is shown in Figure 3e and Table 2. The results showed that polymer concentration influenced the thickness, since F1 (which has only BC) had the lowest thickness (0.04 ± 0.10 mm), while F8 (which had the highest collagen and starch concentration) had the highest thickness (0.11 ± 0.03 mm). Thickness influenced the WVP, which ranged from 5.59 ± 0.44 × 10^−8^ g·mm/m^2^·h·Pa (F1) to 14.06 ± 0.71 × 10^−8^ g·mm/m^2^·h·Pa (F9), with a significant difference (*p* > 0.05) (Figure 3f and Table 2). It is important to highlight that the samples with the highest thickness (F7, F8, and F9) had the highest WVP. Moreover, these samples had the highest collagen concentration (2.00%), indicating that the presence of collagen in the biocomposite formulation can increase not only the WS, as previously mentioned, but also the thickness and WVP.

The grammage of BC–collagen–starch biocomposites was 15.03–338.83% more than that of pure BC (Figure 3g and Table 2). F4 and F8, with 2.23% starch concentration and 1% and 2% collagen concentration, respectively, had the highest grammage (39.58 ± 0.87 and 39.10 ± 0.73 g·cm^−2^, respectively). In addition, the behavior of the samples in relation to the thickness was similar, indicating a correlation between these two properties. However, unlike these properties, the opacity of pure BC (36.67 ± 0.37 Abs 600 nm·mm^−1^) was greater than that of BC–collagen–starch biocomposites (31.85 ± 1.98–8.37 ± 0.08 Abs 600 nm·mm^−1^) (Figure 3h and Table 2). These results are similar to those reported by the visual appearance of the samples (Figure 2), where, after the addition of the biopolymers, the samples obtained showed transparency.

**Figure 4:**
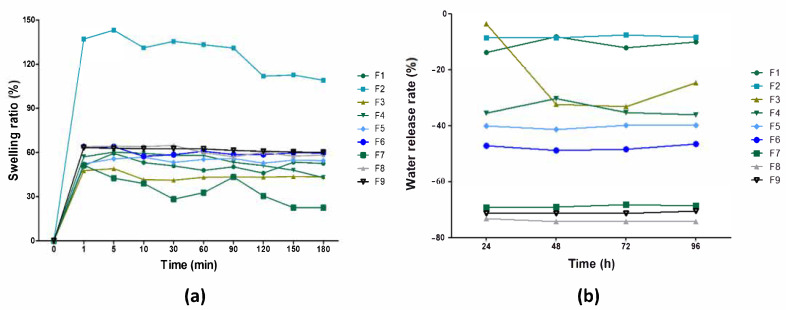
Characterization of pure bacterial cellulose (BC,F1) and BC–collagen–starch biocomposites (F2–F9). (**a**) Swelling rate and (**b**) water retention rate.

The hydrophilic properties of pure BC and the BC–collagen–starch biocomposites over time were analyzed through the swelling assay and WRR, as shown in Figure 4. The swelling rate of the samples ranged between 47.61% and 65.75% after the first minute of analysis (Figure 4a). This increase in the swelling rate was attributed to the effect generated by hydrating the material. In general, the swelling rate was maintained without major oscillations, except for F2 and F7. The swelling rate of F2 (a BC–starch biocomposite) after 1 min was 137.04% (one with the greatest hydrophilic character) and, after 10 min, it decreased to 109.04% until the 180^th^ minute. Similarly, the swelling rate of F7 (a BC–collagen biocomposite) decreased from 51.31% after 1 min to 22.52% after 180 min, a greater loss than that found for the other samples (F1, F3, F4, F5, F6, F8, and F9). The water release capacity reduced in all samples, regardless of the analysis time (Figure 4b). These results indicate that the samples degraded after the period of immersion in water, which can be justified by the biodegradable character of the biopolymers present. In general, the capacity to release water over time was maintained, except in F3, which showed a sharp reduction of approximately 109% compared to the analysis times of 24 and 48 h.

### 3.3. Mechanical Properties of Pure BC and BC–Collagen–Starch Biocomposites

**Figure 5:**
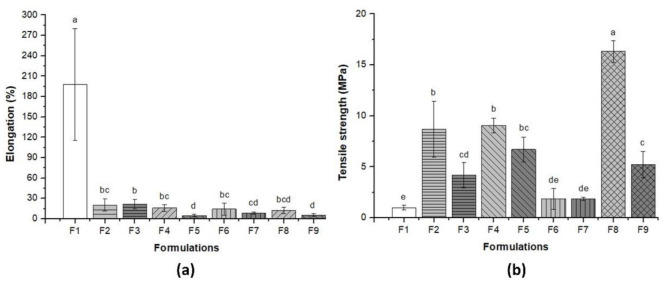
Mechanical properties of pure bacterial cellulose (BC,F1) and BC–collagen–starch biocomposites (F2–F9). (**a**) Elongation (%) and (**b**) tensile strength (MPa).

Figure 5 and Table 2 show the results concerning the elongation at break (Figure 5a) and tensile strength (Figure 5b) of pure BC and BC–collagen–starch biocomposites. F1 (pure BC) had the highest elongation (197.94 ± 82.13%), while after the addition of the other biopolymers, these values decreased by up to 40%, varying between 4.81% and 21.39% for F5 and F3, respectively. However, the tensile strength increased more than 189% after the addition of biopolymers in the cellulose matrix, and ranged from 0.98 ± 0.23 to 16.32 ± 1.04 MPa for F1 and F8, respectively. These results indicate that although a plasticizer (glycerol) was used during sample production, the presence of biopolymers, regardless of their concentration, reduced the elasticity of the biocomposites. Therefore, BC–collagen–starch biocomposites, particularly F8, which had the highest concentration of polymers in its constitution, showed greater rigidity than pure BC.

### 3.4. Morphological Property of Pure BC and BC–Collagen–Starch Biocomposites

**Figure 6:**
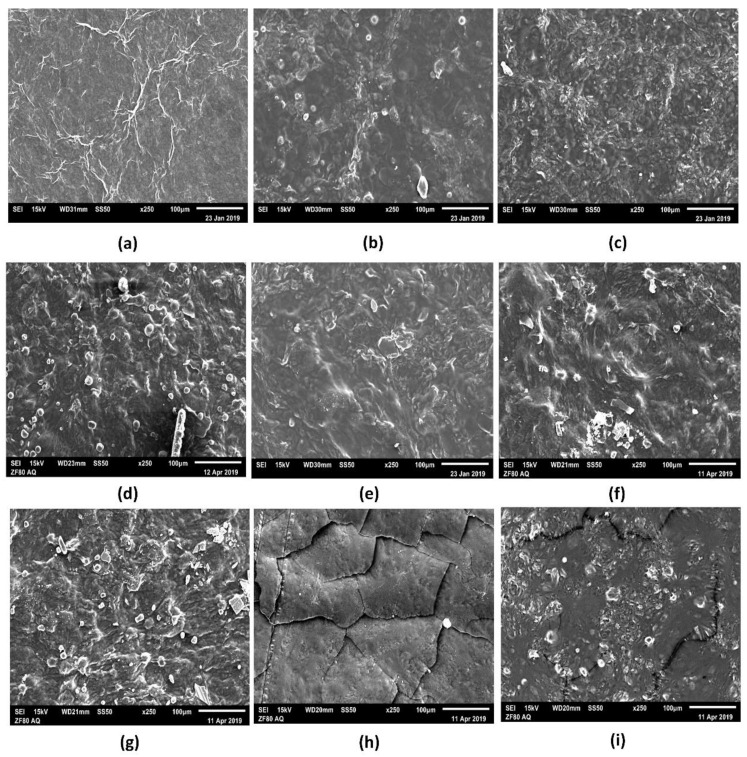
Scanning electron microscopy (SEM) surface micrographs of the surfaces of the pure bacterial cellulose (BC, F1) and BC–collagen–starch biocomposite (F2–F9). (**a**) F1, (**b**) F2, (**c**) F3, (**d**) F4, (**e**) F5, (**f**) F6, (**g**) F7, (**h**) F8, and (**i**) F9.

Figure 6 shows the micrographs of the nine samples produced, highlighting the structural details. Figure 6a, referring to the pure BC (F1), shows that the porous structure formed by the cellulose fibers, a fundamental characteristics of BC, could be observed on the surface; however, the thickness of individual fibers could not be determined at this magnitude. Due to the addition of the biopolymers, and consequently the formation of biocomposites, the characteristic fibrous network of BC is not apparent (Figure 6b–i). This behavior indicates that there is a structural interaction between the BC matrix, collagen, and starch, which may have changed the visual appearance of the samples from opaque in pure BC to transparent in biocomposites (Figure 1). Thus, the micrographs obtained indicate that the formation of biocomposites may promote surface compaction. Nevertheless, this compaction caused micro-fractures in biocomposites with increased concentrations of polymers, as shown in the micrographs of F8 and F9 (Figure 6h,i, respectively). In addition, it is noted that air microbubbles were present in the biocomposite structure (Figure 6b–g,i), which must have been derived during the casting process.

### 3.5. Thermal Property of Pure BC and BC–Collagen–Starch Biocomposites

**Figure 7:**
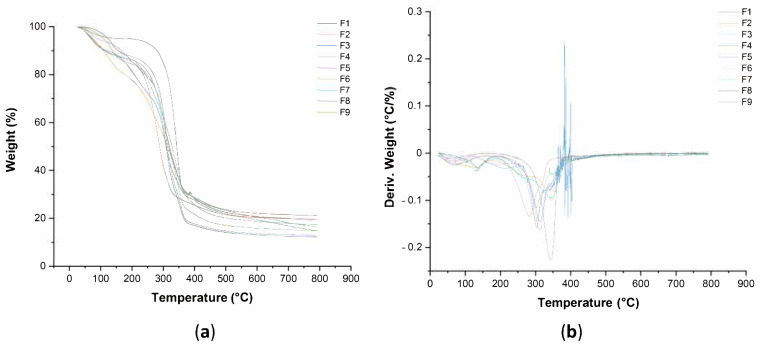
Thermal analysis of pure bacterial cellulose (BC,F1) and BC–collagen–starch biocomposites (F2–F9). (**a**) Thermogravimetric analysis (TGA) and (**b**) differential thermogravimetry (DTG).

To determine the stability and thermal behavior of pure BC and the BC–collagen–starch biocomposites, the TGA (Table S1) and DTG curves were determined as shown in Figure 7a,b, respectively (Figure 7). In general, the thermal decomposition of the nine samples showed three stages of mass loss. The first stage refers to water evaporation, that is, moisture loss, which occurred between 25 and 250 °C, where the loss in mass varied between 5 and 20% for F1 and F6, respectively. However, pure BC showed a higher temperature range in this stage than the BC–collagen–starch biocomposites. The second stage of thermal degradation occurred between 200 and 350 °C and may be associated with the degradation of proteins and polyhydroxylated compounds (cellulose fibers). The loss in mass of the samples at this stage was approximately 59%, with F8 and F9, which had the highest polymer concentration in their composition, showing the greatest losses of approximately 62%. Therefore, this stage was the one with the highest mass loss rate, since it corresponded to the degradation of BC, collagen, starch, and glycerol. In addition, it is responsible for the peaks observed in the DTG curve between the temperatures of 300 and 350 °C. The third stage initiated between 300 and 350 °C and extended up to 800 °C in association with a loss in mass between 78% for F1 and 84% for F8 and F9. Normally, this stage is associated with the subsequent decomposition of the residua, leading to the formation of inorganic matter and carbonaceous char.

### 3.6. Effect of the Presence of Components on the Properties of BC–Collagen–Starch Biocomposites: Principal Component Analysis

**Figure 8:**
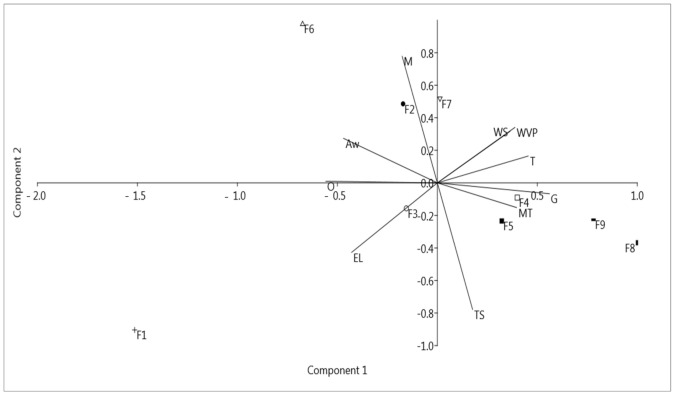
Scores scatter plot by principal component analysis of pure bacterial cellulose (BC,F1) and BC–collagen–starch biocomposites (F2–F9).

To assess the effect of the presence and concentration of biopolymers on the produced biocomposites, PCA was performed with physical and barrier properties data, except SR and WRR, and the elongation and tensile strength assays (Figure 8). PC1 explained 49.54% of the total variance of the data, while PC2 explained 27.07%, thus explaining 76.61% of the cumulative variance. The highest loadings for PC1 were grammage and tensile strength, while those for PC2 were moisture and WVP. In general, Figure 8 shows that the BC–collagen–starch biocomposites showed a tendency to group according to the presence and concentration of their constituents, whereas pure BC (F1) did not form a cluster with any other sample analyzed. However, it is important to highlight that the BC–starch (F2 and F3) and BC–collagen (F6 and F7) biocomposites were allocated in different quadrants, indicating that the biopolymer concentration results in a biocomposite with different properties. In addition, it was noted that some variables such as moisture content and TS content were negatively correlated to each other. The graph of scores obtained through PCA analysis also showed that the presence and concentration of the biopolymers analyzed in the BC matrix influenced their properties.

## 4. Discussion

Wound care has historically been one of the most basic and essential practices of human civilization [4]. Currently, an increasing amount of information about the effectiveness of traditional and technologically advanced practices is available [63,64,65]. Many strategies are being developed to increase the efficiency of the healing process by considering the different phases of the tissue repair process and the particularities of each injury type [66,67,68,69]. The nine samples analyzed showed different behaviors in relation to the type of test performed, and this variation occurred according to their polymeric constitution.

One of the differences observed was in relation to the visual appearance, where F1 (pure BC) was primarily opaque, while the BC–collagen–starch biocomposites (F2–F9) were comparatively more transparent. The opacity of F1 was 22.85% higher than that of F8, which had the lowest opacity. The opacity of BC has already been demonstrated to be related to the presence of an ultrafine nanofibrous network [56,70], which forms a dense crosslinked structure stabilized by hydrogen bonds and is highly crystalline (between 60 and 90%) [71,72]. Within this context, BC nanofibers have low transparency and reflect light. Furthermore, the high crystallinity of BC may have influenced opacity, since the crystalline regions may act by reflecting or diverting the incident light beam, which may compromise its transmission, resulting in increased opacity [56]. Thus, the addition of collagen and/or starch and the interaction between these biomaterials and cellulose nanofibers may have altered the crystallinity, resulting in a less opaque and more transparent biocomposite. Abral et al. [73] showed that the opacity of biocomposites formed by cassava starch, polyvinyl alcohol (PVA), and BC increased according to the BC fiber concentration in the polymeric blend. Wilpiszewska et al. [74] observed that films based on carboxymethylated derivatives of starch and cellulose were transparent and elastic; however, Santos et al. [75] demonstrated that biocomposites of thermoplastic corn starch and BC showed a lower transparency than pure BC, different results from those found in our study. It is important to highlight that using a transparent wound dressing allows the patient to assess the wound healing process continuously without stimulating the injured area, thus reducing the probability of secondary injuries, particularly in cases of dermal lesions [76]. Thus, the application of samples F2–F9 in clinical practice is highly relevant.

Aiming at the application as a wound dressing, it is fundamental to understand whether the material formed has a hydrophobic or hydrophilic character as well as to know the a_w_ in its structure. The a_w_ of the nine samples analyzed ranged from 0.400 to 0.480, which is within the ideal range (0.2–0.6) proposed by Cázon et al. [77], since the results of their study showed that biocomposites of BC, PVA, and chitosan did not decrease the WPV, one of the main properties that an ideal wound dressing should exhibit. In addition, the study demonstrated that a_w_ varied according to the composition of the biocomposite, similar to the results found in that study [77]. Based on the results, it was also observed that starch and collagen incorporation in BC altered the properties related to WS as well as to moisture and TS. WS is directly related to the increase in the hydroxyl group in polar polymeric matrices, which enhances hydrogen bond formation with water, thus forming soluble materials that facilitates its biodegradability [78]. The use of biodegradable materials in biomedicine is necessary, as it traditionally uses non-biodegradable polymer-based materials such as petrolatum gauze as wound dressings, which can affect the environment. Thus, the use of biocomposites based on BC, collagen, and starch can be considered as an environment friendly alternative. With regard to moisture, F6 (BC–collagen biocomposite) had the highest moisture, which differed from the results reported by Pasaribu et al. [79], which pointed to the decrease in moisture content in BC after collagen incorporation.

Saska et al. [80] showed that although the incorporation of collagen in the BC membrane slightly increased the swelling rate compared to that of pure BC, the study by Noh et al. [81] showed that increasing the concentration of BC in biocomposites of BC-collagen enhanced the water uptake capacity. Priya et al. [82] observed that the incorporation of cellulose fibers in the starch–PVA matrix decreased the swelling rate. The results reported in the studies by Saska et al. [80] and Priya et al. [82] were similar to those found in our study, where the presence of collagen did not significantly alter the swelling rate, while F2 (with the highest starch concentration without collagen) had the highest swelling rate. The swelling rate assesses the absorption of water or aqueous fluids by the analyzed material without dissolving, and this swelling continues until there is a balance between the water and the material [83]. Due to its swelling rate, F2 has the potential to be applied to lesions caused by burns, since the main consequences of its healing physiology are the high release of exudates [84]. The stability of the water release capacity of pure BC and the biocomposites based on collagen and starch, with the exception of F3, indicates that they can contribute to the maintenance of adequate moisture in the lesion microenvironment, favoring tissue regeneration and preventing bacterial proliferation [18,85].

Vapor exchange through a material is a critical property directly related to the effectiveness of wound dressing. Our results showed that increasing collagen concentration (2.00%) increased the WPV; however, it is important to note that the presence of starch also contributed to the high permeability of F8 and F9. This behavior was similar to that reported by Zhuang et al. [86], who observed that starch composites and tilapia skin collagen had a higher WPV than that in the film composed only of collagen. Nevertheless, the study by Tibolla et al. [87] showed that the starch–cellulose nanocomposites with a higher concentration of cellulose fibers had lower values for WPV. High WVP promotes wound dehydration and helps in inducing scar formation, while low WVP can slow down the wound healing process due to the deposition of the high amount of exudates. Therefore, a suitable dressing should display an optimal WVP value [88].

Grammage is a property that is directly related to the mechanical resistance and barrier properties of the material, since they are influenced by the thickness and mechanical properties [56]. F8, with the highest polymer concentration in its constitution, had the highest grammage, thickness, and consequently, tensile strength, while F1, with only BC in its composition, had the lowest grammage, thickness, and consequently, the highest elongation at break. It is important to note that the SEM micrographs of F1 and F8 (Figure 6a,h) did not show air microbubbles, which may have impacted their mechanical performance. The thickness found in this study was similar to those of films based on BC, glycerol, and PVA as reported by Cazón et al. [89] The authors also reported that the thickness varied between 0.02 and 0.105 mm, being directly proportional to the amount of polymer present in the film produced. Cazón et al. [89] further reported that the tensile strength increased according to the increase in thickness, as reported in this study. Another study showed that tensile strength as well as the Young’s modulus of nanocomposites based on starch and cellulose from banana increased, according to the concentration of cellulose nanofibers in the composition [87]. Qin et al. [90] demonstrated that the tensile strength of collagen-cellulose nanocrystals films increased as the cellulose content increased, where films with 7% and 10% of cellulose nanocrystals presented 1.22 ± 0.36 MPa and 1.57 ± 0.19 MPa, respectively, while the tensile strength of the control sample (only with collagen in its composition) was 0.45 ± 0.19 MPa. The results reported by Antosik et al. [91] showed a different behavior from those found in our study, where the increase in starch concentration resulted in a greater elongation at break and, consequently, a lower tensile strength. The difference between the two studies may be associated with the type of cellulose used, since the study by Antosik et al. [91] used carboxymethyl cellulose of plant origin. An ideal material for wound dressing should preferably have a high elongation at break, since the mechanical stretching of the material may induce tissue regeneration [92,93]. This study showed that F1 (pure BC) had a high elongation (169.54%), while F2 and F3 (BC–starch biocomposite) had the highest elongation (20.29 and 21.39%, respectively) among the biocomposites produced. It should also be noted that F1 was the only biocomposite without a plasticizer (glycerol) in its constitution, which could have resulted in less elongation. However, this result indicates that the compatibility between the biopolymers present in the biocomposite was high, which facilitated their interaction and, consequently, the formation of strong chemical bonds. The elongation of the BC membrane was also superior in the study of Lee et al. [94] compared to that of the membrane consisting only of collagen.

As shown in Figure 7, the thermal stability of the samples also varied according to their constitution. The results showed that F1 had the highest thermal stability. The thermal stability of BC is mainly associated with high crystallinity, high water content, and high purity [95]. Martins et al. [96] reported an increased thermal stability in starch films with the addition of BC, and attributed this increase to not only the high thermal stability of cellulose, but also the excellent polymeric compatibility between starch and cellulose. However, Zhijiang and Guang [38] reported that the thermal stability of the composite improved and the temperature of thermal degradation increased with the incorporation of collagen compared to that of pure BC. Moraes et al. [97] observed that the thermal stability of the BC in the presence of collagen decreased by 30 °C. Nevertheless, the authors considered that BC–collagen hydrogels showed excellent thermal stability. This difference between the studies may be related to the acetylation effect reported by the authors [38], since acetic acid was used as a solvent for collagen, which may have resulted in cellulose acetylation, a process that may increase thermal stability.

## 5. Conclusions

In the present study, nine different samples were produced based on BC, collagen, starch, and glycerol as the plasticizing agents, with fixed masses of BC and glycerol. All samples were characterized based on their physical, barrier, morphological, mechanical, and thermal properties. The results obtained showed that the performance of a sample was directly related to the presence and the concentration of its constituent components. The different performances indicate that pure cellulose as well as biocomposites may have different application potentials, mainly depending on the injury type and stage of healing. Thus, the results of this study can contribute to a new perspective in biomedicine (i.e., the treatment of injuries with high-performance biomaterials) positively impacting the quality of life of patients.

## Figures and Tables

**Table 1:** Sample name and composition of biocomposites based on bacterial cellulose and combined with starch and collagen.

Formulation (Sample Name)	Biocomposite Composition (%, m·v^−1^)			
	Bacterial Cellulose	Collagen	Starch	Glycerol
F1	50.00	–	–	–
F2	50.00	–	2.23	0.60
F3	50.00	–	1.12	0.60
F4	50.00	1.00	2.23	0.60
F5	50.00	1.00	1.12	0.60
F6	50.00	1.00	–	0.60
F7	50.00	2.00	–	0.60
F8	50.00	2.00	2.23	0.60
F9	50.00	2.00	1.12	0.60

**Table 2:** Data regarding the physical properties, barrier, and mechanical properties of pure bacterial cellulose (sample F1) and bacterial cellulose–collagen–starch biocomposites (samples F2–F9).

Analysis	Sample Name								
	F1	F2	F3	F4	F5	F6	F7	F8	F9
Water activity	0.48 ^a^ ± 0.01	0.44 ^b^ ± 0.06	0.45 ^b^ ± 0.01	0.45 ^b^ ± 0.02	0.40 ^c^ ± 0.01	0.48 ^a^ ± 0.01	0.48 ^a^ ± 0.01	0.42 ^c^ ± 0.01	0.42 ^c^ ± 0.01
Moisture (%)	10.88 ^c^ ± 0.64	19.34 ^a^ ± 1.64	13.06 ^bc^ ± 1.67	13.69 ^bc^ ± 1.05	13.37 ^bc^ ± 0.88	20.60 ^a^ ± 0.69	15.66 ^b^ ± 0.04	11.19 ^c^ ± 0.81	10.75 ^c^ ± 0.89
Total solids (%)	89.12 ^a^ ± 0.64	80.66 ^c^ ± 1.64	86.94 ^ab^ ± 1.67	86.31 ^ab^ ± 1.05	86.63 ^ab^ ± 0.88	79.4 ^c^ ± 0.69	84.34b ± 0.04	88.81 ^a^ ± 0.81	89.25 ^a^ ± 0.89
Water solubility (%)	12.94 ^e^ ± 2.58	27.11 ^d^ ± 6.52	31.12 ^cd^ ± 2.31	36.76 ^cd^ ± 1.62	41.79 ^bc^ ± 2.02	52.33 ^b^ ± 1.74	69.67 ^a^ ± 12.7	54.12 ^b^ ± 2.21	69.76 ^a^ ± 1.43
Water vapor permeability (10^−8^ g·mm/m^2^·h·Pa)	5.59 ^e^ ± 0.44	10.01 ^d^ ± 0.51	10.18 ^d^ ± 0.38	10.89 ^cd^ ± 0.33	10.19 ^d^ ± 0.57	11.94 ^bcd^ ± 0.61	13.37 ^ab^ ± 1.52	12.66 ^abc^ ± 0.45	14.06 ^a^ ± 0.71
Opacity (Abs 600 nm·mm^−1^)	36.67 ^a^ ± 0.37	9.49 ^ef^ ± 0.12	8.82 ^f^ ± 0.04	11.12 ^d^ ± 0.05	12.03 ^d^ ± 0.64	31.85 ^b^ ± 1.98	16.02 ^c^ ± 0.52	8.37 ^f^ ± 0.08	9.17 ^ef^ ± 0.81
Grammage (g·cm^−2^)	8.91 ^e^ ± 0.46	17.57 ^d^ ± 0.21	10.25 ^e^ ± 0.19	39.58 ^a^ ± 0.87	26.25 ^c^ ± 0.84	16.41 ^d^ ± 0.69	24.66 ^c^ ± 3.97	39.10 ^a^ ± 0.73	33.11 ^b^ ± 0.52
Thickness (mm)	0.04 ^d^ ± 0.10	0.08 ^c^ ± 0.01	0.09 ^bc^ ± 0.01	0.10 ^b^ ± 0.02	0.08 ^c^ ± 0.01	0.08 ^c^ ± 0.01	0.09 ^c^ ± 0.02	0.11 ^a^ ± 0.03	0.10 ^b^ ± 0.01
Elongation (%)	169.54 ^a^ ± 6.00	20.29 ^b^c ± 6.35	21.39 ^b^ ± 5.76	16.07 ^bcd^ ± 5.04	4.81 ^d^ ± 2.0	14.29 ^bcd^ ± 3.22	8.61 ^cd^ ± 1.67	10.55 ^bcd^ ± 1.75	5.57 ^d^ ± 1.94
Tensile strength (Mpa)	0.99 ^e^ ± 0.23	8.7 ^b^ ± 2.74	4.17 ^cd^ ± 1.23	9.05 ^b^ ± 0.75	6.72^bc^ ± 1.22	1.5 ^de^ ± 0.76	1.8 ^de^ ± 0.15	16.32^a^ ± 1.04	5.21 ^c^ ± 1.31

Values showing the same letter (a, b, c, d, e or f) in the same column do not show significant difference (*p* < 0.05) through the Tukey test at a 95% confidence level (Statistical analysis).

## Data Availability

Data is contained within the article or supplementary materials.

## Supplementary Materials

The following are available online at https://www.mdpi.com/1996-1944/14/2/458/s1, Figure S1: Real images of bacterial cellulose production and biocomposites, Table S1: Main values related to the thermogravimetric analysis of pure bacterial cellulose (sample F1) and bacterial cellulose–collagen–starch biocomposites (samples F2–F9).
