# Supplementary material for: Development of Bacterial Cellulose Biocomposites Combined with Starch and Collagen and Evaluation of Their Properties
Source: Materials (Basel). 2021 Jan 19;14(2):458. doi: 10.3390/ma14020458 (PMC7833372; doi:10.3390/ma14020458)
Supplement: Supplementary file 1 [file materials-14-00458-s001.pdf]

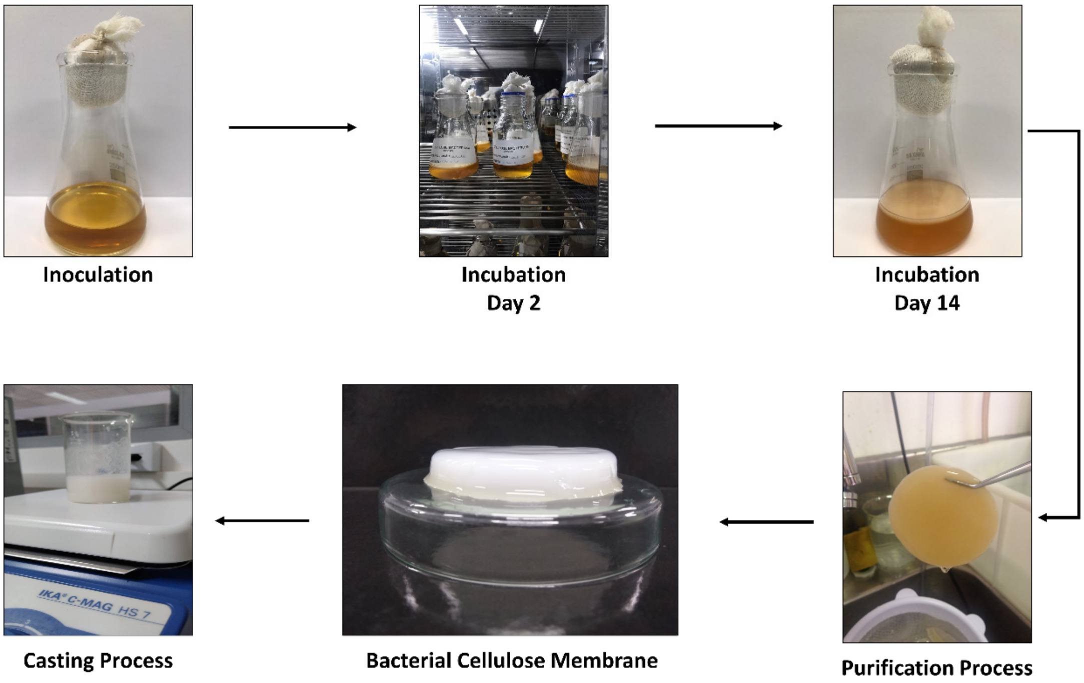

Figure S1. Real images of bacterial cellulose production and biocomposites.

Table S1. Data regarding the physical properties, barrier, and mechanical properties of pure bacterial cellulose (sample F1) and bacterial cellulose–collagen–starch biocomposites (samples F2-F9).

**Analysis**	**Sample Name**								
	**F1**	**F2**	**F3**	**F4**	**F5**	**F6**	**F7**	**F8**	**F9**
Water activity	0.48^{a}±0.01	0.44^{b}±0.06	0.45^{b} ± 0.01	0.45^{b} ± 0.02	0.40^{c} ± 0.01	0.48^{a} ± 0.01	0.48^{a} ± 0.01	0.42^{c} ± 0.01	0.42^{c} ± 0.01
Moisture (%)	10.88^{c}±0.64	19.34^{a}±1.64	13.06^{bc}±1.67	13.69^{bc}±1.05	13.37^{bc}±0.88	20.60^{a}±0.69	15.66^{b}±0.04	11.19^{c}±0.81	10.75^{c}±0.89
Total solids (%)	89.12^{a}±0.64	80.66^{c}±1.64	86.94^{ab}±1.67	86.31^{ab}±1.05	86.63^{ab}±0.88	79.4^{c}±0.69	84.34^{b}±0.04	88.81^{a}±0.81	89.25^{a}±0.89
Water solubility (%)	12.94^{e}±2.58	27.11^{d}±6.52	31.12^{cd}±2.31	36.76^{cd}±1.62	41.79^{bc}±2.02	52.33^{b}±1.74	69.67^{a}±12.7	54.12^{b}±2.21	69.76^{a}±1.43
Water vapor permeability (10^{-8} g.mm/m^{2}.h.Pa)	5.59^{e}±0.44	10.01^{d}±0.51	10.18^{d}±0.38	10.89^{cd}±0.33	10.19^{d}±0.57	11.94^{bcd}±0.61	13.37^{ab}±1.52	12.66^{abc}±0.45	14.06^{a}±0.71
Opacity (Abs 600nm. mm^{-1})	36.67^{a}±0.37	9.49^{ef}±0.12	8.82^{f}±0.04	11.12^{d}±0.05	12.03^{d}±0.64	31.85^{b}±1.98	16.02^{c}±0.52	8.37^{f}±0.08	9.17^{ef}±0.81
Grammage (g.cm-^{2})	8.91^{e}±0.46	17.57^{d}±0.21	10.25^{e}±0.19	39.58^{a}±0.87	26.25^{c}±0.84	16.41^{d}±0.69	24.66^{c}±3.97	39.10^{a}±0.73	33.11^{b}±0.52
Thickness (mm)	0.04^{d}±0.10	0.08^{c}±0.01	0.09^{bc}±0.01	0.10^{b}±0.02	0.08^{c}±0.01	0.08^{c}±0.01	0.09^{c}±0.02	0.11^{a}±0.03	0.10^{b}±0.01
Elongation (%)	169.54^{a}± 6.00	20.29^{bc}±6.35	21.39^{b}±5.76	16.07^{bcd}±5.04	4.81^{d}±2.0	14.29^{bcd}±3.22	8.61^{cd}±1.67	10.55^{bcd}±1.75	5.57^{d}±1.94
Tensile strength (Mpa)	0.99^{e}±0.23	8.7^{b}±2.74	4.17^{cd}±1.23	9.05^{b}±0.75	6.72^{bc}±1.22	1.5^{de}±0.76	1.8^{de}±0.15	16.32^{a}±1.04	5.21^{c}±1.31

Table S2. Main values related to thermogravimetric analysis of pure bacterial cellulose (sample F1) and bacterial cellulose–collagen–starch biocomposites (samples F2–F9).

Sample name	Temperature range (°C)	Weight loss range (%)
**F1**	29.58 – 250.03	0 – 6.93
	250.03 – 350.92	6.93 – 60.02
	350.92 – 791.26	60.02 – 78.84
**F2**	26.55 – 246.98	0 – 23.56
	246.98 – 347.90	23.56 – 71.72
	347.90 – 788.22	71.72 – 80.59
**F3**	29.09 – 249.51	0 – 27.62
	249.51 – 350.41	27.62 – 73.77
	350.41 – 790.73	73.77 – 87.77
**F4**	23.55 – 244.29	0 – 17.62
	244.29 – 345.05	17.62 – 64.66
	345.05 – 785.42	64.66 – 83.51
**F5**	26.46 – 250.31	0 – 21.25
	250.31 – 351.20	21.25 – 72.24
	351.20 – 791.35	72.24 – 87.21
**F6**	24.19 – 247.28	0 – 30.31
	247.28 – 348.02	30.31 – 66.66
	348.02 – 788.14	66.66 – 85.28
**F7**	28.15 – 244.91	0 – 20.53
	244.91 – 345.80	20.53 – 59.71
	345.80 - 785.91	59.71 – 82.8
**F8**	25.06 – 248.78	0 – 16.13
	248.78 – 349.66	16.13 - 63.60
	349.66 – 789.80	63.60 – 80.61
**F9**	29.58 – 245.75	0 – 19.74
	245.75 – 346.63	19.74 – 62.31
	346.63 – 786.83	62.31 – 85.24
